# PP2A inhibition from LB100 therapy enhances daunorubicin cytotoxicity in secondary acute myeloid leukemia via miR-181b-1 upregulation

**DOI:** 10.1038/s41598-017-03058-4

**Published:** 2017-06-06

**Authors:** Chao Hu, Mengxia Yu, Yanling Ren, Kongfei Li, Dominic M. Maggio, Chen Mei, Li Ye, Juying Wei, Jie Jin, Zhengping Zhuang, Hongyan Tong

**Affiliations:** 1 Department of Hematology, The First Affiliated Hospital, College of Medicine, Zhejiang University, Hangzhou, 310003 People’s Republic of China; 2 Department of Hematology, Hangzhou First People’s Hospital, Hangzhou, 310006 Zhejiang Province People’s Republic of China; 3 Myelodysplastic Syndromes Diagnosis and Therapy Center, The First Affiliated Hospital, College of Medicine, Zhejiang University, Hangzhou, 310003 People’s Republic of China; 4 Department of Hematology, Yin Zhou People’s Hospital, Ningbo, 315040 Zhejiang Province People’s Republic of China; 5 Surgical Neurology Branch, National Institute of Neurological Disorders and Stroke, National Institutes of Health, Bethesda, MD 20892 USA

## Abstract

Patients with secondary acute myeloid leukemia (sAML) arising from myelodysplastic syndromes have a poor prognosis marked by an increased resistance to chemotherapy. An urgent need exists for adjuvant treatments that can enhance or replace current therapeutic options. Here we show the potential of LB100, a small-molecule protein phosphatase 2 A (PP2A) inhibitor, as a monotherapy and chemosensitizing agent for sAML using an *in-vitro* and *in-vivo* approach. We demonstrate that LB100 decreases cell viability through caspase activation and G2/M cell-cycle arrest. LB100 enhances daunorubicin (DNR) cytotoxicity resulting in decreased xenograft volumes and improved overall survival. LB100 profoundly upregulates miR-181b-1, which we show directly binds to the 3′ untranslated region of Bcl-2 mRNA leading to its translational inhibition. MiR-181b-1 ectopic overexpression further diminishes Bcl-2 expression leading to suppression of sAML cell growth, and enhancement of DNR cytotoxicity. Our research highlights the therapeutic potential of LB100, and provides new insights into the mechanism of LB100 chemosensitization.

## Introduction

The myelodysplastic syndromes (MDS) are a group of hematological disorders characterized by hematopoietic progenitor cells with dysplastic cell morphology, ineffective hematopoiesis, and potential for clonal evolution^1^. MDS represent the most common cause of acquired bone marrow failure in adults, and up to 30% of patients progress to secondary acute myeloid leukemia (sAML)^2–5^. Evolution to late stage MDS involves upregulation of anti-apoptotic proteins such as Bcl-2, and downregulation of pro-apoptotic proteins such as Fas and Myc^6–8^. Transformation to sAML has been linked to inactivation of tumor suppressive genes such as p53 and p15^lnk4b^ ^9,10^. Collectively these changes result in a diminished ability for cell cycle control, and contribute to the aggressive phenotype and chemoresistant behavior typified by sAML^5^. More effective therapeutic strategies are urgently needed to help patients afflicted with this grave condition.

Protein phosphatase 2 A (PP2A) is a highly conserved dual-specificity phosphatase that plays a pivotal role in regulating cell cycle protein activity and inhibition of apoptosis through direct interaction with serine/threonine phosphorylation switches^11–13^. It is often seen with elevated activity and/or expression in neoplastic cells where it functions as a positive regulator of cell growth and survival^14–16^. PP2A promotes resistance to apoptosis through direct dephosphorylation of Bcl-2^17^, and through dephosphorylative activation of the inhibitory kinase of caspase-2, CaMKII^18^. PP2A is a positive regulator of Ras/Raf/MEK/ERK signaling, an anti-apoptotic pathway well characterized in states of malignant transformation^19–23^. Targeted inhibition of PP2A in p53 overexpressing HeLa cells has been shown to induce cell cycle arrest at least partially through increased levels of the Cdk5 activator, p25. Upregulated Cdk5 in turn facilitates Bax translocation into the mitochondrial membrane to promote apoptosis^24^. Similarly, PP2A inhibition of T leukemia cells has been demonstrated to result in caspase-dependent apoptosis through p38 MAPK activation and loss of mitochondrial transmembrane potential^25^. PP2A inhibition in human myeloid cell lines induces cell cycle arrest and apoptosis through increased degradation of Bcl-2 mRNA, although the direct mechanism of transcript destabilization has not yet been seen^26–29^. PP2A inhibition has shown promise in the treatment multiple tumor types including glioma, sarcoma, pancreatic cancer and del(5q) MDS^30–33^. Hence, targeting PP2A may be a potential strategy in sAML chemotherapy.

Pharmacologic inhibition of PP2A has generally been studied using a variety of naturally produced, but toxic molecules. Okadaic acid is a PP1 and PP2A inhibitor produced by dinoflagellates presumably as a cytotoxic self-defense agent^34^. Although it exhibits potent apoptotic effects in many human cancer cell lines^35–37^, its neurotoxic and enterogenic effects limit its use^38,39^. Cantharidin is an odorless organic chemical secreted by the blister beetle used for more than 2000 years in traditional Chinese medicine to treat a variety of disorders including MCV infections and warts^40^. Cantharadin is a selective PP2A inhibitor that induces cell-cycle arrest and apoptosis in a variety of cancer subtypes such as breast, colon, pancreatic, hepatocellular, and bladder carcinoma^41–49^. Nevertheless, cantharidin is associated with severe side effects due to high gastrointestinal and renal toxicity^50,51^. Researchers have recently focused on LB100, a synthetic cantharidin with specific PP2A inhibitory activity that does not appear to exhibit significant systemic toxicity^32,52,53^. LB100 has shown promising anti-neoplastic activity as a solo chemotherapy agent, and also as a radio- and chemotherapy sensitizer against glioblastoma, pheochromocytoma, breast cancer, nasopharyngeal cancer, hepatocellular carcinoma, pancreatic cancer, and ovarian cancer^31,33,52–58^. It has also shown synergistic cytotoxic effects with doxorubicin to inhibit progression of stem cell-derived aggressive sarcoma^32^. As such, it is currently in Phase I clinical trials as a potential treatment against progressive and metastatic solid tumors^59^, with another phase I clinical trial planned for the treatment of low-risk MDS resistant to lenalidomide^30^. However, LB100 has not yet been studied in models of sAML, and its mechanism of chemosensitization has not been directly elucidated.

Here we investigate the activity of LB100 against sAML as a monotherapy and chemosensitizing agent in conjunction with daunorubicin, a standard therapeutic used to treat AML. We explored its mechanism of action and chemosensitization in depth using multiple AML and sAML cell lines, and verified our findings in an *in-vivo* sAML mouse xenograft.

## Results

### LB100 attenuates PP2A activity and reduces sAML cell viability

**Figure 1:**
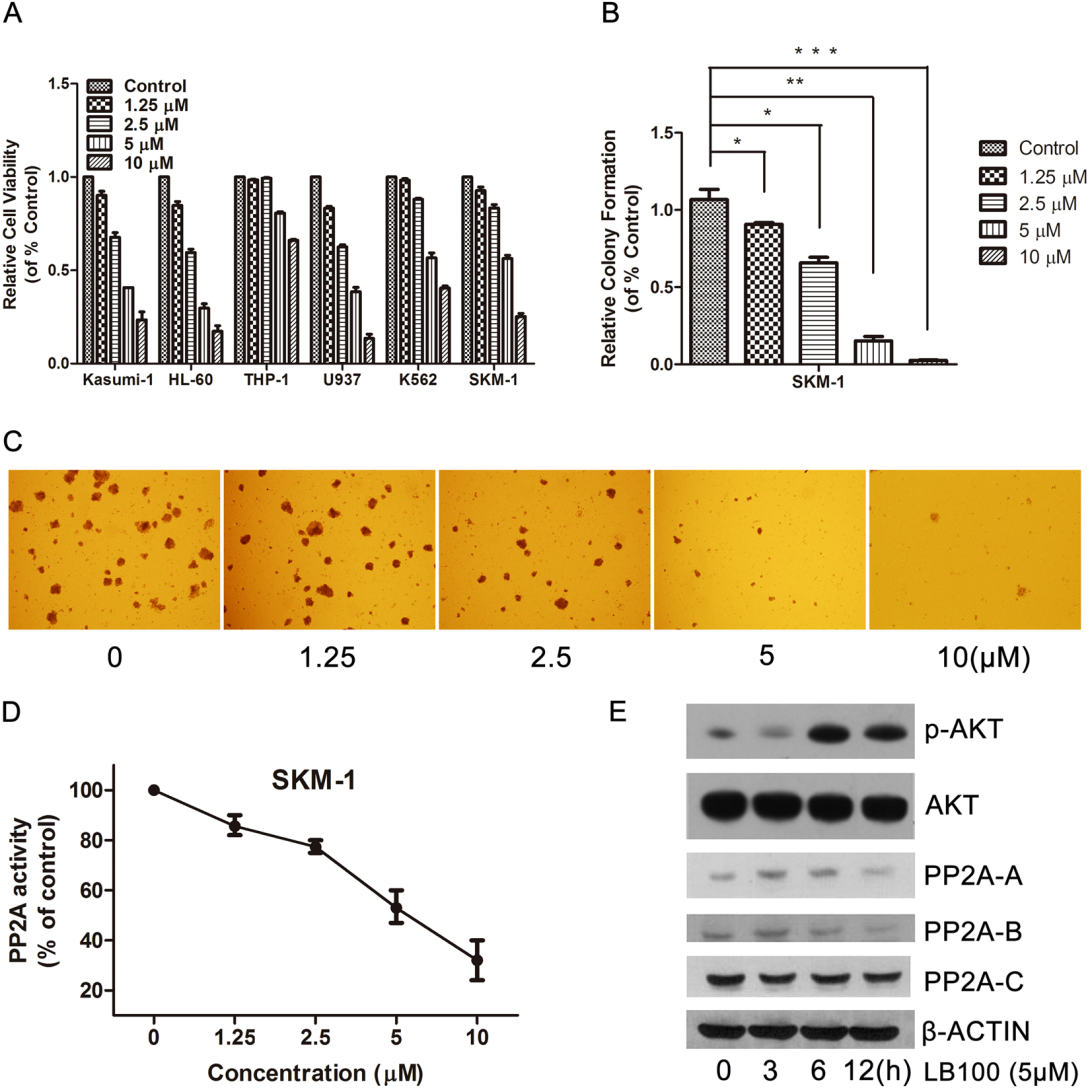
Inhibition of PP2A by LB100 decreases AML and sAML cell proliferation. (**A**) LB100 decreased cell proliferation in multiple leukemia cell lines in a dose dependent manner. (**B**,**C**) SKM-1 colony formation rate significantly decreased following LB100 treatment in a concentration dependent fashion after 7 days of culture in methylcellulose medium. Images were obtained at x40 magnification. (**D**) After 6 hours of LB100 treatment, PP2A activity was decreased with increasing concentrations of LB100 in SKM-1 cells. (**E**) PP2A isoform levels were moderately decreased after receiving 5 μM LB100 treatment for 12 h. Statistically significant differences are marked by an asterisk (*P < 0.05; **P < 0.01, ***P ≤ 0.001).

To examine LB100 cytotoxicity we evaluated cell viability in 6 different leukemia cell lines including the sAML cell line: SKM-1. Each cell line was determined using an MTT cytotoxicity assay where a linear concentration-dependent cytotoxicity plot for LB100 was seen in all tested cell lines. The IC50 values for Kasumi-1, HL-60, THP-1, U937, K562 and SKM-1 at 24 h after treatment were 4.38, 3.36, 13.46, 3.44, 7.00 and 5.35 μM, respectively (Fig. 1A). Interestingly, we found that LB100 exhibited profound cytotoxic activity not only in AML cell lines, but also in the sAML cell line. The dose-dependent inhibitory activity of LB100 on the growth of SKM-1 cells was further confirmed by colony formation assays (Fig. 1B,C).

Previous studies have shown that LB100 can reduce PP2A activity in several kinds of solid tumors^52,57^. Consistent with these findings, exposure to 10 μM LB100 for 12 hours reduced the activity of PP2A up to 60% in SKM-1 cells (Fig. 1D). Moreover, LB100 moderately decreased the expression of the three PP2A subunits (PP2A-A, PP2A-B, and PP2A-C) in sAML cells, as confirmed by western blot method (Fig. 1E). LB100 also increased levels of p-AKT, which is expected since AKT is a direct substrate of PP2A. These results confirm that LB100 effectively inhibits sAML cell growth possibly through PP2A inhibition.

### LB100 triggers G2/M phase arrest in sAML cells through modulation of cell cycle regulatory proteins

**Figure 2:**
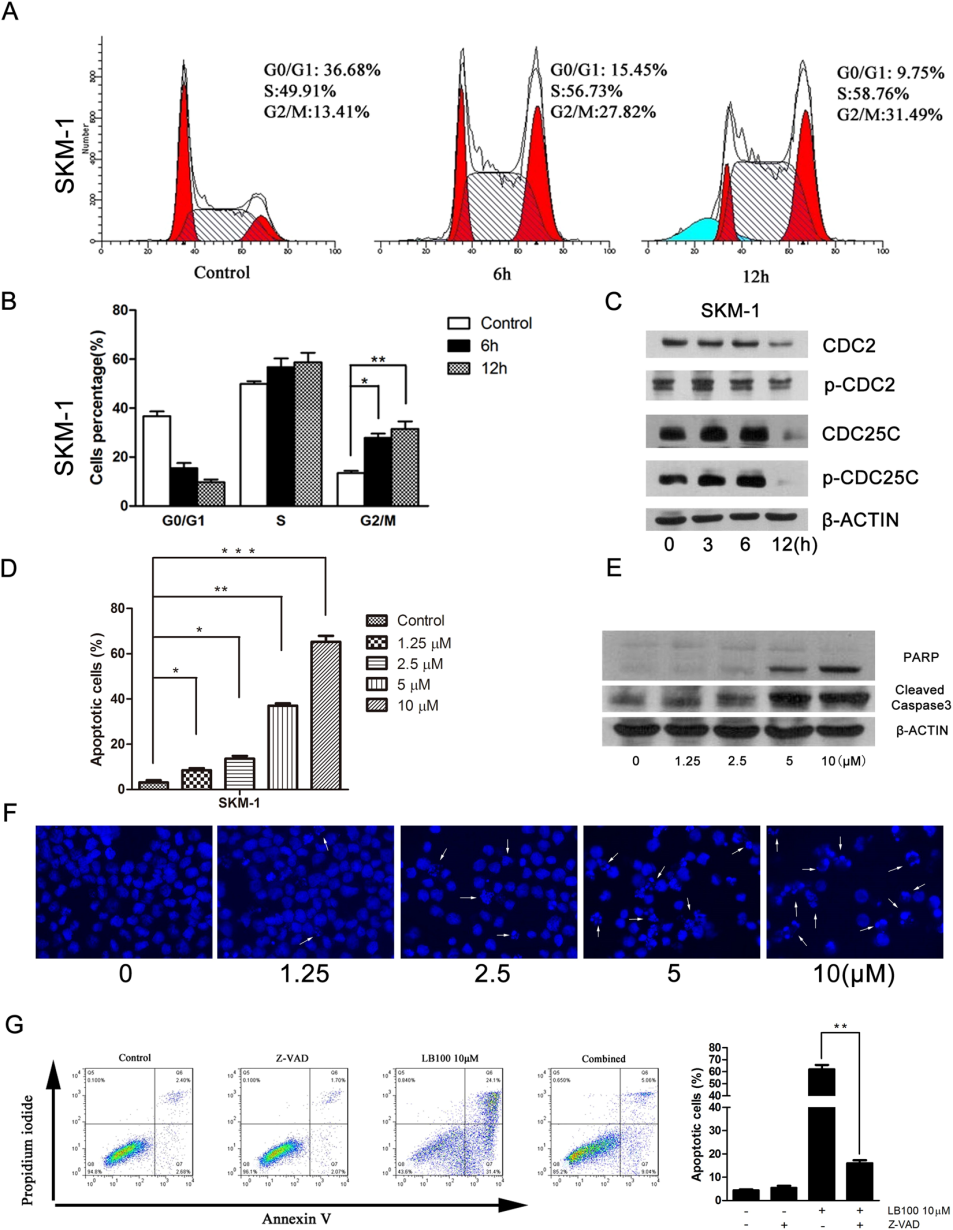
Analysis of cell cycle changes and apoptosis after LB100 treatment. (**A**,**B**) Flow cytometry analysis was performed to determine the relative percentage of SKM-1 cells in various phases of the cell cycle after 5 μM LB100 treatment for 0, 6 and 12 h. Quantification of data demonstrates a time-dependent shift towards G2/M phase. (**C**) SKM-1 cells displayed a time-dependent decrease in G2/M regulatory proteins after 5 μM LB100 treatment. (**D**) Flow cytometry analysis of SKM-1 cells stained with annexin V and propidium iodide demonstrated LB100 induced apoptosis in a concentration-dependent manner. (**E**) SKM-1 cells displayed a dose dependent increase in cleaved caspase 3 and PARP after 24 h exposure to LB100. (**F**) Fluorescent microscopy of Hoechst-stained SKM-1 cells demonstrated an increased amount of condensed and/or fragmented nuclei after progressively high doses of LB100. (**G**) Flow cytometry analysis was performed to quantify the proportion of apoptotic cells after 24 h of LB100 treatment (10 μM) or control, in the presence or absence of z-VAD-FMK. z-VAD-FMK rescued cells from LB100-induced cell death, demonstrating a caspase-dependent mechanism of LB100-mediated cytotoxicity. Statistically significant differences are marked by an asterisk (*P < 0.05; **P < 0.01, ***P ≤ 0.001).

The underlying mechanism for LB100-mediated tumor suppression was further investigated through analysis of changes in cell-cycle behavior and protein expression. Flow analysis of SKM-1 cells demonstrated that 12 h exposure to LB100 at 5 μM dramatically decreased G0/G1 (from 36.7% to 9.8%), and significantly increased G2/M phase cells (from 13.4% to 31.5%) (Fig. 2A,B). The accumulation of G2/M phase cells occurred in a time-dependent manner. Consistent with these findings, the G2-to-M checkpoint molecules CDC2 and CDC25C were markedly downregulated in terms of both total- and phosphorylated-protein levels (Fig. 2C). This is in agreement with previous studies investigating LB100 function^52,54,60^. Our findings suggest that LB100 attenuated sAML cell growth at least partly from induction of mitotic cell arrest.

### LB100 induces apoptotic cell death in sAML cells

To determine the influence of apoptosis on the observed decreases in cell proliferation after LB100 administration, we used an Annexin V and Propidium Iodide labeled flow-cytometry assay. LB100 demonstrated a concentration-dependent increase in the fraction of apoptotic sAML cells from 3.13% in the absence of LB100, to 8.51%, 13.61%, 37%, and 65.27% in the presence of 1.25 μM, 2.5 μM, 5 μM and 10 μM of LB100, respectively (Fig. 2D). This finding was confirmed with microscopic analysis of sAML cells after Hoechst staining identified increased amounts of condensed, pyknotic nuclei (Fig. 2F). Immunoblotting also demonstrated LB100-induced caspase-3 and PARP cleavage in a concentration-dependent manner (Fig. 2E). The effect of pan-caspase inhibition using z-VAD-FMK on LB100-induced apoptosis was also studied. The inhibitor partially blocked LB100-induced apoptosis, decreasing the rate of apoptosis from 62% to 16% (Fig. 2G). Collectively, our findings suggest that LB100 decreased sAML proliferation at least partly from inducing cellular apoptosis.

### LB100 augments daunorubicin-mediated tumoricidal effects

**Figure 3:**
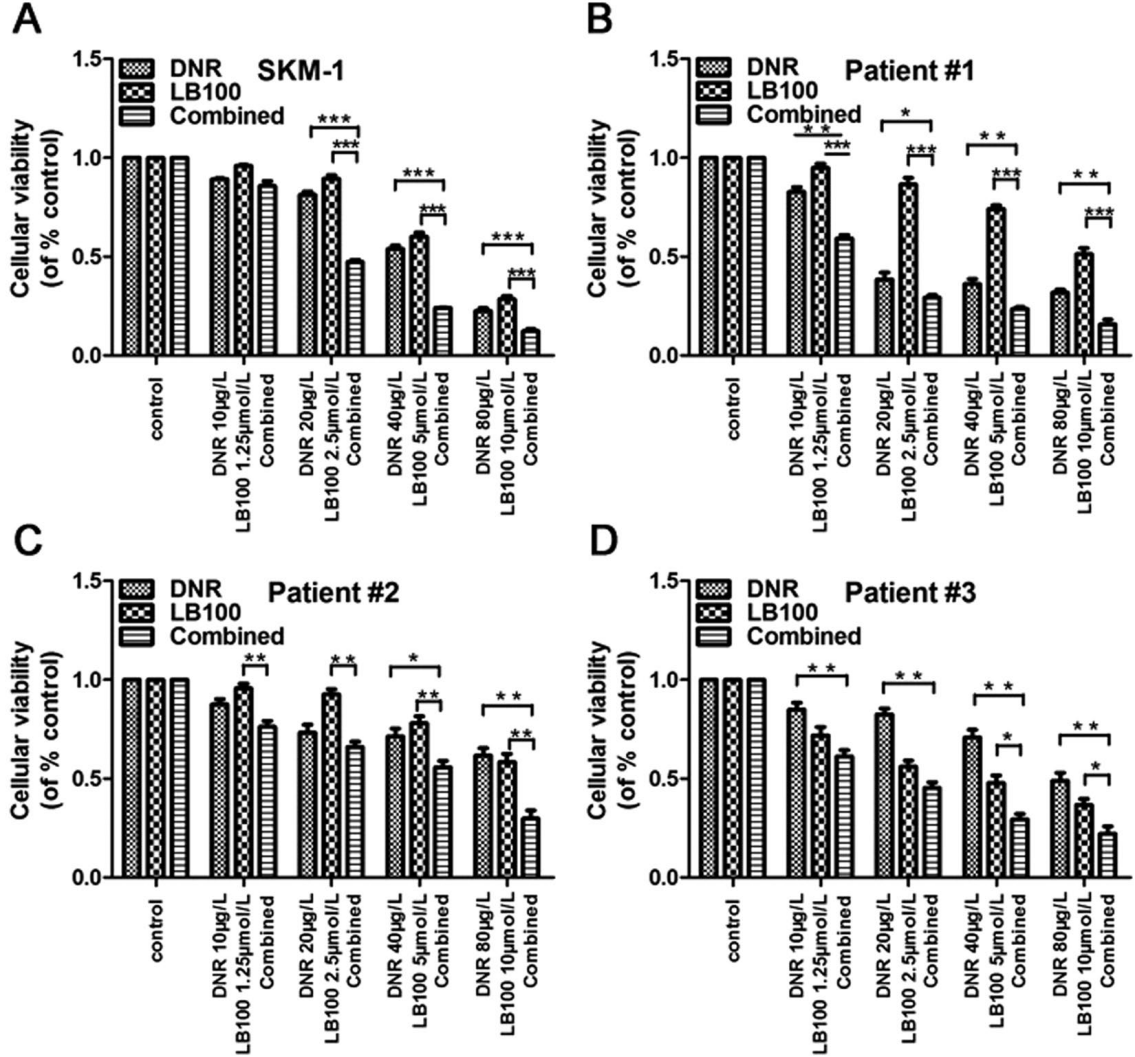
LB100 enhances daunorubicin cytotoxicity to sAML *in vitro*. (**A–D**) LB100 significantly enhanced the cytolytic activity of daunorubicin in SKM-1 cells, and in three primary sAML patient samples. Statistically significant differences are marked by an asterisk (*P < 0.05; **P < 0.01, ***P ≤ 0.001).

The chemosensitization potential of LB100 was studied using an *in-vitro* and *in-vivo* approach to determine whether the tumoricidal effects of daunorubicin (DNR), a common therapeutic agent used in patients with sAML, could be synergistically increased by combinatorial treatment. SKM-1 cell viability was significantly diminished in a dose-dependent manner following 24 h incubation with either LB100 or DNR. Simultaneous treatment with LB100 and DNR dramatically reduced SKM-1 cell viability compared to monotherapy with either agent (Fig. 3A). The addition of LB100 similarly sensitized sAML patients’ bone marrow mononuclear cells to DNR treatment (Fig. 3B–D). A one- to five-fold increase in the AML suppression ratio was seen in each patient cohort with LB100 and DNR co-treatment.

**Figure 4:**
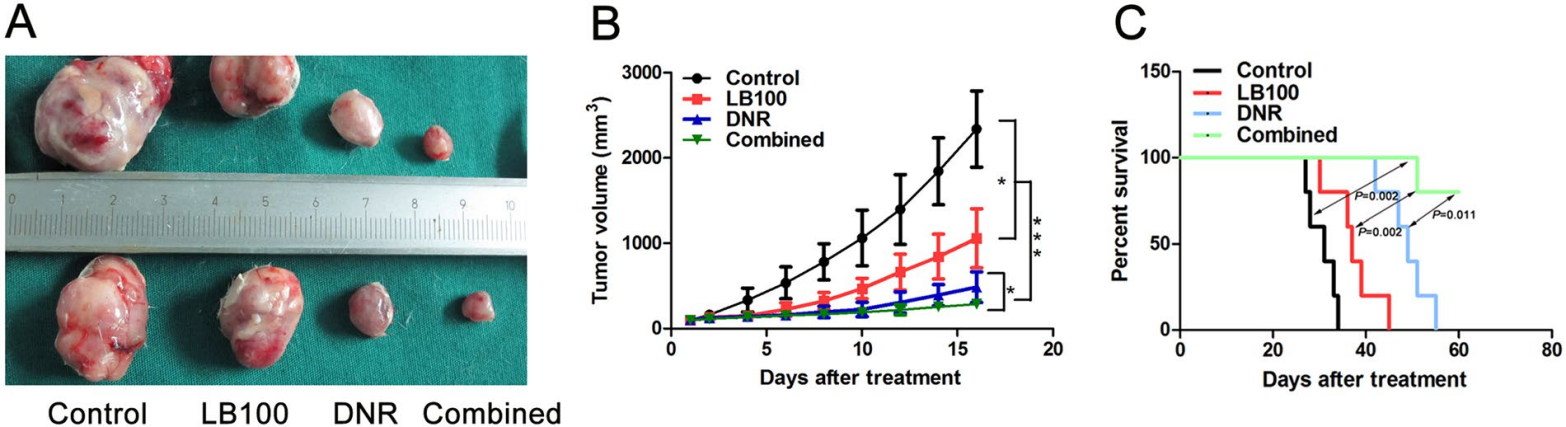
LB100 in combination with daunorubicin significantly decreases tumor burden *in vivo*. (**A**,**B**) Tumor volume was significantly lower in mice receiving LB100 (*P* = 0.017) or Daunorubicin monotherapy (*P* < 0.001) as compared to control. Further benefit was seen with combination daunorubicin + LB100 therapy as compared to mice receiving either monotherapy, or control. (**C**) A significant improvement in overall survival was observed with daunorubicin + LB100 therapy versus control (*P* = 0.002), versus daunorubicin monotherapy (*P* = 0.011), and versus LB100 monotherapy (*P* = 0.002). Statistically significant differences are marked by an asterisk (*P < 0.05; ***P ≤ 0.001).

SKM-1 xenografts demonstrated a sharp decrease in tumor volume after receiving either LB100 (*P* = 0.017) or DNR monotherapy (*P* < 0.001) alone as compared to control. Mice receiving the combination therapy of LB100 plus DNR had further decreases in tumor volume as compared with control or either monotherapy (Fig. 4A,B, control *P* < 0.001, LB100 *P* < 0.001, DNR *P* < 0.05). Mice in the combination treatment group were also found to have a significantly prolonged overall survival (Fig. 4C, vs control *P* = 0.002, vs LB100 *P* = 0.002, vs DNR *P* = 0.011). Our findings demonstrate a synergistic tumoricidal effect with concurrent LB100 and DNR administration. This is in agreement with other studies reporting simultaneous PP2A inhibition enhancing the efficacy of chemotherapy treatment for solid tumors^31,32^.

### LB100 facilitates sAML chemosensitivity through miR-181b-1 upregulation

**Figure 5:**
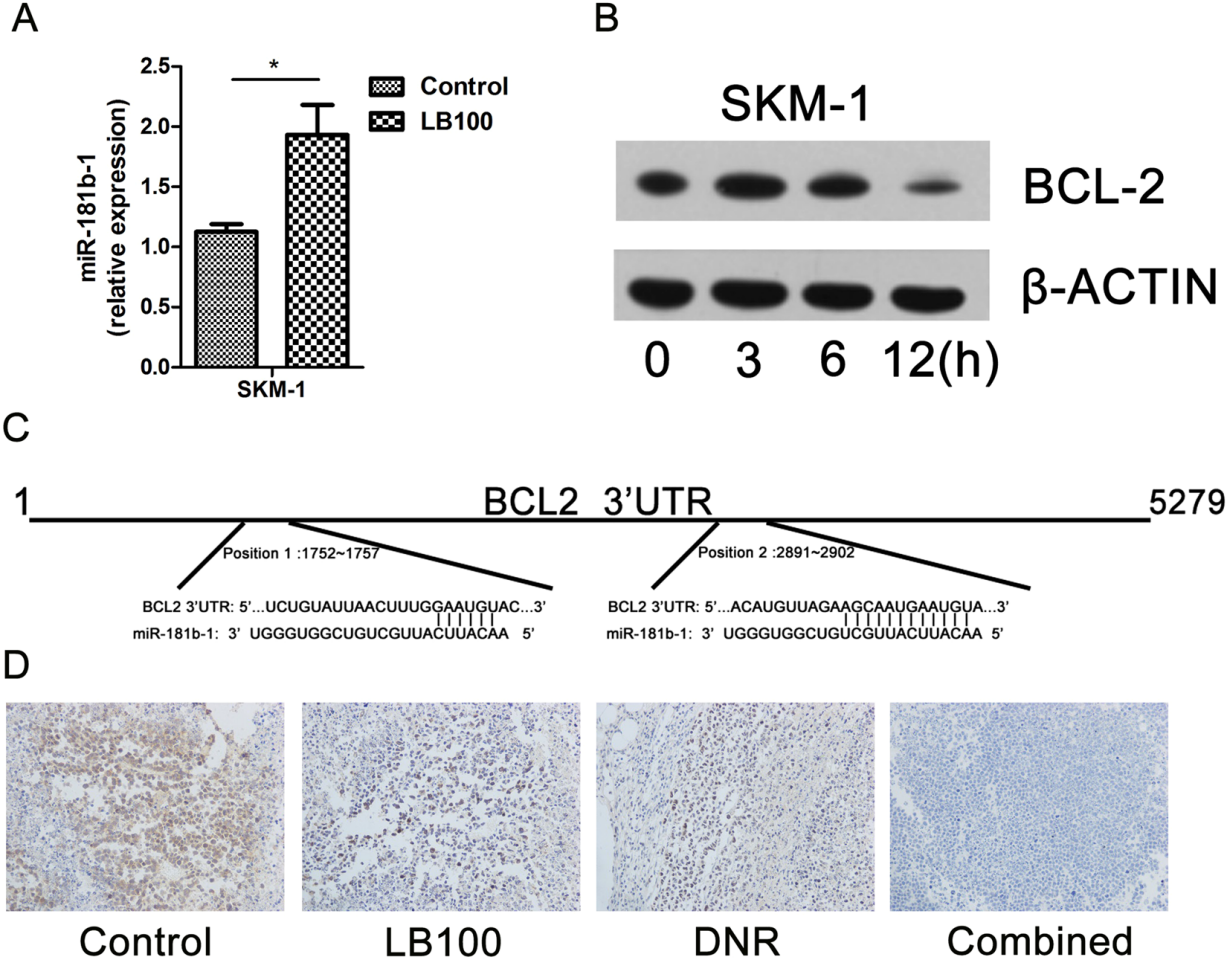
LB100 induces expression of miR-181b-1, and suppresses expression of Bcl-2. (**A**) qRT-PCR demonstrated that LB100 upregulated miR-181b-1 in SKM-1 cells. (**B**) Western blot demonstrated decreased Bcl-2 expression after 5 μM of LB100 treatment for 0, 3, 6 and 12 h. β-ACTIN served as an internal control. (**C**) TargetScan predicted miR-181b-1 sequence complementarity to the 3′ untranslated region of Bcl-2 mRNA. (**D**) SKM-1 xenograft histology demonstrates decreased immunoreactivity to Bcl-2 after LB100 treatment. Statistically significant differences are marked by an asterisk (*P < 0.05).

The mechanism underlying LB100 chemosensitization was investigated by assessing the epigenetic response of sAML to LB100 administration. miRNAs are endogenous 17–25 base pair noncoding RNA molecules that play prominent regulatory roles in malignant transformation, stem cell maintenance, metastasis, and invasiveness^61–67^. MicroRNA profiling was performed to analyze the SKM-1 transcriptome for differences after LB100 administration (5 μM, exposure 12 h). miR-181b-1 was found to be significantly up-regulated in the LB100 treatment group. Interestingly, miR-181b-1 has previously been identified as an important mediator of cisplatin and vincristine chemosensitivity in human gastric and lung cancer cell lines^68^. qRT-PCR was then performed to confirm upregulation of miR-181b-1 between the LB100 treatment and control group. After normalization to an endogenous control (U6 RNA), the relative expression of miR-181b-1 was found to be increased about 2 fold after LB100 treatment (*P* = 0.049) (Fig. 5A).

To identify putative targets of miR-181b-1, we utilized the online miRNA prediction software TargetScan to screen transcripts with a 3′ untranslated region (UTR) containing a similar sequence complementarity as miR-181b-1. The Bcl-2 mRNA transcript was identified among the potential targets with a 3′UTR containing two highly conserved 8-mer sites complementary to the seed region of the miR-181b-1 (Fig. 5C). Considering the well-characterized anti-apoptotic function of Bcl-2, we hypothesized that miR-181b-1 may play an important role in the chemosensitization potential of LB100 by facilitating cell death through inhibition of Bcl-2 translation. Bcl-2 expression was analyzed via immunoblot and immunohistochemistry in *in-vitro* and *in-vivo* models, respectively, and was found to be markedly downregulated after LB100 administration (Fig. 5B,D).

**Figure 6:**
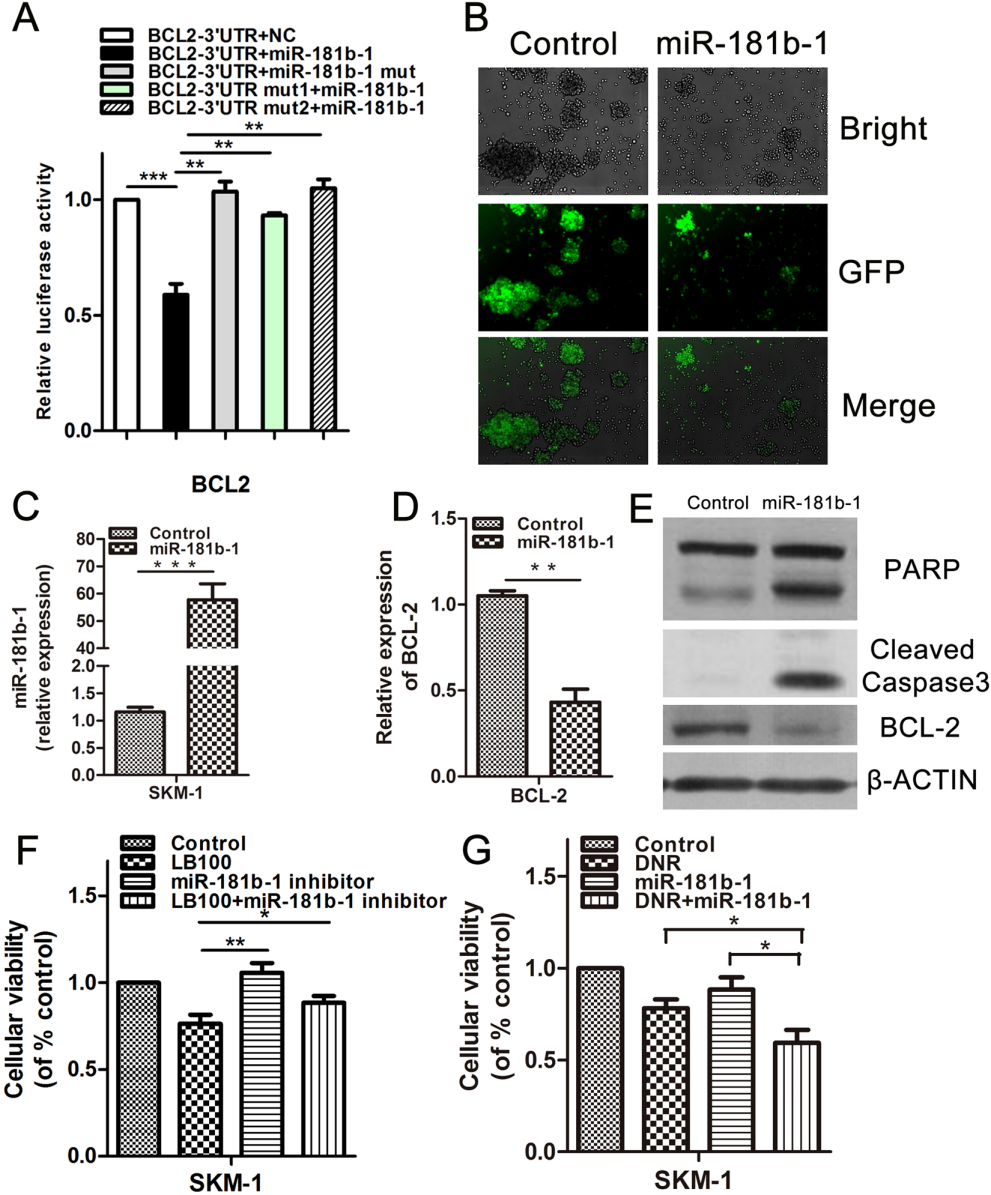
miR-181b-1 directly targets Bcl-2 to mediate chemosensitization of sAML cells to DNR. (**A**) Dual luciferase assay demonstrates a significant decrease in relative luciferase activity in 293T cells when pMIR-REPORT-Bcl-2-3′UTR was coinfected with miR-181b-1 retrovirus, but not with normal control (NC) or mut-miR-181b-1. Mutation of the miR181b-1 binding sites on the Bcl-2 3′UTR additionally did not demonstrate changes in luciferase activity after administration of miR-181b-1 retrovirus. (**B**) GFP was checked after SKM-1 cells were stably infected with miR-181b-1 retrovirus. (**C**–**E**) Overexpression of miR-181b-1 reduced Bcl-2 mRNA and protein levels in SKM-1 cells, and induced expression of cleaved caspase 3. (**F**) Reversal of LB100 (2.5 μM) induced SKM-1 cell death was noted after administration of anti-miRNA targeting miR-181b-1 (20 nM). (**G**) Overexpression of miR-181b-1 significantly enhanced the cytotoxic activity of DNR in sAML cells. Statistically significant differences are marked by an asterisk (*P < 0.05; **P < 0.01, ***P ≤ 0.001).

Dual luciferase assays were utilized to confirm whether miR-181b-1 directly interacted with the 3′UTR of the Bcl-2 mRNA transcript. The 3′UTR of Bcl-2 was cloned downstream of firefly luciferase using a pMIR-REPORT vector. Normal control (empty vector), miR-181b-1, mutant miR-181b-1, and 3′UTR of Bcl-2 binding sites mutant vectors were also utilized. Significant suppression of luciferase activity by miR-181b-1 was observed, which was not seen in the other groups (Fig. 6A). Ectopic miR-181b-1 overexpression (Fig. 6B,C) greatly decreased Bcl-2 mRNA (Fig. 6D) and protein levels in SKM-1 cells (Fig. 6E). Additionally, we found overexpression of miR-181b-1 to mimic the function of LB100 by activating the caspase cascade, inhibiting cell proliferation, and enhancing DNR cytotoxicity (Fig. 6G). Administration of anti-miRNA specific to miR-181b-1 to SKM-1 cells exposed to LB100 significantly reversed the degree of cell death due to LB100 (Fig. 6F). These results suggest that LB100 sensitizes sAML cells to DNR therapy by inducing miR-181b-1 upregulation, causing a subsequent downregulation of Bcl-2.

## Discussion

Patients with myelodysplastic syndromes that secondarily evolve into acute myelogenous leukemia have a median survival of only 15 months despite best standard of care treatment^5^. sAML is characteristically resistant to aggressive induction/consolidation chemotherapy regimens including concomitant cytarabine + daunorubicin. A major mechanism of oncogenic chemoresistance involves overexpression of aberrant anti-apoptotic proteins such as Bcl-2^69–72^. Indeed, overexpression of Bcl-2 has been shown to accelerate tumorigenesis in transgenic mice, and is notably overexpressed in various diseases including malignant hematonosis^70,73^. Bcl-2 is an essential intracellular protein that prevents apoptosis by controlling mitochondrial membrane permeability, preventing the release of pro-apoptotic mitochondrial factors such as cytochrome c, halting induction of downstream caspases, and maintaining mitochondrial function^74–77^. Its overexpression results in an inability of the intrinsic apoptotic pathway to mediate cell death, rendering a distinct survival advantage to mutagenized cells^78^. Downregulation of the Bcl-2 oncoprotein can restore the apoptotic pathway and resensitize malignant cells to the effects of therapy-induced apoptosis. Recent studies have reported reversal of chemoresistance using an antisense approach to target Bcl-2 in models of chronic lymphocytic leukemia, non-Hodgkins lymphoma, and multiple myeloma^79–82^. However, few studies have similarly investigated methods to overcome chemoresistance in models of AML and sAML. In the present study we examined the chemosensitizing potential of LB100, a small-molecule inhibitor of PP2A, in a preclinical model of AML and sAML.

We found that LB100 suppressed AML and sAML cell proliferation, and enhanced the chemotherapeutic efficacy of daunorubicin (DNR) in sAML cells by halting the cell cycle and facilitating apoptosis. These effects were observed across multiple cell lines, and were recapitulated in a mouse sAML xenograft. To determine the mechanism for chemosensitization, we explored the epigenetic response of the sAML cell line to LB100 treatment. MicroRNAs (miRNAs) have been recently suggested as endogenous master regulators of protein expression^83^. Their differential expression is seen in various disease states, and has the potential to cause or propagate pathophysiologic cell processes^84,85^. They have been implicated in providing malignant cells their chemoresistant abilities^86–89^, and are aberrantly expressed in various subtypes of AML^90–93^. We identified miR-181b-1 as significantly upregulated in sAML cells after treatment with LB100. Increased levels of miR-181b-1 have been correlated with improved overall survival in patients with cytogenetically normal, and cytogenetically abnormal AML^91,94–96^. Recently, Lu *et al*. showed that miR-181b-1 was downregulated in the chemoresistant human leukemia cell lines K562/A02 and HL60/ADM compared to parental K562 and HL-60 cells^97^. Restoration of miR-181b-1 was noted to sensitize K562/A02 and HL-60/Ara-C cell lines to doxorubicin and cytarabine by targeting HMGB1. To determine the function of miR-181b-1 in sAML, we performed an in-silico analysis using TargetScan to search for miR-181b-1 putative targets based on the complementary 3′ UTR base pair sequence. Bcl-2 was identified as a top prospective target based on sequence complementarity. Consistent with our microarray results, we found a marked increase in miR-181b-1 levels via qRT-PCR in SKM-1 cells treated with LB100. Bcl-2 levels were correspondingly downregulated in the SKM-1 cell line, as well as in an SKM-1 NOD-SCID mouse xenograft. A gain-of-function study was conducted in the sAML cell line through transfection with a retrovirus causing overexpression of miR-181b-1. Dual luciferase assay demonstrated miR-181b-1 directly interacting with the Bcl-2 transcript’s 3′ UTR, with qRT-PCR and immunoblotting demonstrating an associated decrease in Bcl-2 mRNA and protein expression. Furthermore, administration of anti-miR-181b-1 rescued sAML cells from LB100’s cytotoxic effects. We investigated the effects of miR-181b-1 overexpression in the setting of concurrent DNR administration and found a significant augmentation of DNR sAML cytolytic activity. This is in line with our prior results demonstrating LB100-mediated chemosensitivity of DNR to sAML cells. Taken together, our findings suggest that LB100 suppresses sAML cell proliferation, and sensitizes sAML cells to DNR chemotherapy at least partly due to upregulation of miR-181b-1, which in turn downregulates Bcl-2 through direct translational inhibition. We believe this to be a novel mechanism of how LB100 augments sAML cell chemosensitivity.

The exact manner underlying how LB100 induces upregulation of miR-181b-1 is still yet to be discovered. In general, the mechanisms behind proteomic regulation of epigenetic molecules is relatively unknown. One report identified a feedback loop involving PP2A, AKT, MYC, and miR-29a, wherein the PP2A substrate MYC was shown to directly suppress miR-29a in a model of AML^98^. Another study demonstrated that knockdown of the PP2A substrate eukaryotic initiation factor 4E (eIF4E) caused a dramatic decrease in miR-134, miR-199b, and miR-424 expression in a model of melanoma^99^. Conversely, eIF4E overexpression had the opposite effect of increasing the expression of the mentioned miRNAs. It is possible that stimulating G2/M cell arrest through inhibition of PP2A leads to upregulation of miR-181b-1 to promote apoptosis of cells through downregulation of Bcl-2. The abnormal microtubule configuration at the metaphase plate in cells given selective PP2A inhibitors^100^, might serve as a distress signal indicating the non-viability of the cell. Further experiments are needed to investigate the sequential actions involving LB100 upregulation of miR-181b-1.

The dephosphorylation of CDC2 and CDC25C after selective PP2A inhibition has previously been noted by other investigators^101^. Prior to the onset of mitosis, there is a highly regulated balance between cyclinB-Cdc2 and PP2A^102–104^. The balance dictates the phosphorylation level of mitotic substrates, including CDC2 and CDC25C, and is essential to allow the correct entry into and exit from mitosis^105–107^. At baseline, PP2A is held in a state of incomplete activation by its regulator Greatwall^101^. We found that LB100 administration results in a dose- and time-dependent inactivation of PP2A (Fig. 1D,E), resulting in the time-dependent dephosphorylation of CDC2 and CDC25C (Fig. 2C). It is unknown why CDC2 and CDC25C are degraded with LB100, however other studies have seen similar results with PP2A-specific inhibition^101^. The ubiquitin proteosomal system is intimately associated with these mitotic substrates, and it is possible that alterations of their phosphorylation status might promote their ubiquitin-dependent degradation. These findings are in line with previous results from our group demonstrating G2-M cell cycle arrest after selective PP2A inhibition^32^.

PP2A is a complex molecule that is often targeted for activation in models of malignancy due to its occasional tumor-suppressive properties. FTY720 is a PP2A activator that has shown promising results in preclinical models of AML. To explain this finding, differences in baseline expression of PP2A need to be accounted. Cell lines most responsive to FTY720 have a specific D816V mutation in the tyrosine kinase domain of c-kit^108,109^. This mutation causes decreased basal expression of PP2A, reduced PP2A activity, and higher baseline activation of the Ras/Raf/MEK/ERK signaling cascade^109^. Increased activation of the Ras/Raf/MEK/ERK signaling pathway is known to be associated with malignant transformation of pre-cancerous cells^110^. Administration of FTY720 to AML cells with the D816V mutation is associated with decreased expression of Ras/Raf/MEK/ERK, and decreased cell viability^108,109^. The AML/sAML cell lines utilized in our study differed in that they had low baseline expression of the Ras/Raf/MEK/ERK pathways, along with relatively higher levels of PP2A. Our study demonstrated evidence of pro-apoptotic processes after LB100 administration such as decreased Bcl-2 expression, increased cleaved caspase 3 levels (Figs 2E and 5B), and increased phosphorylated Bcl-2 and CamKII (Supplementary Fig. S1). PP2A is known to promote resistance to apoptosis through dephosphorylative activation of CaMKII^18^. The phosphorylation of Bcl-2 can manifest as a pro-apoptotic signal^111,112^. And PP2A is known to inhibit apoptosis by dephosphorylating Bcl-2 in various tumor cell lines^17^. We utilized Annexin V and propidium iodide FACS analysis (Fig. 2D,G) to demonstrate increased apoptosis after PP2A inhibition in sAML cells. Interestingly, we found increased activation of the anti-apoptotic Ras/Raf/MEK/ERK signaling cascade in the same sAML cell line (Supplementary Fig. S1). This interesting finding is in accordance with our group’s previous investigation involving transformed mesenchymal stem cells (rTDMCs) in a model of aggressive sarcoma^32^. As with many types of AML and sAML, the rTDMC cell line does not demonstrate a baseline inhibition of PP2A. The intrinsic differences in baseline oncogenic signaling pathways might explain the differences in susceptibility to PP2A inhibition vs activation.

In summary, we demonstrate that LB100 has therapeutic potential in the treatment of sAML. As a monotherapy it evokes apoptosis and cell cycle arrest in sAML cells. It synergizes with DNR to provide enhanced sAML cytotoxicity. We provide evidence that LB100 induces upregulation of miR-181b-1 to suppress the proapoptotic protein Bcl-2. We believe these findings provide preclinical support for testing LB100 as an adjunct to DNR to overcome sAML multi-drug resistance.

## Materials and Methods

### Reagents

LB100 was provided by Lixte Biotechnology Holdings, Inc (East Setauket, NY, USA). A stock solution of LB100 (10 mM) was prepared in phosphate-buffered saline (PBS, KeYi, Hangzhou, China) and kept at −80 °C. Daunorubicin (DNR) was purchased from Haizheng Pharmacia (Zhejiang, China) and stored at −80 °C. miR-181b-1 inhibitor was purchased from JiMa (Shanghai, China).

### Established cell lines and primary cell culture

The following five human leukemia cell lines were obtained from the Shanghai Institute of Cell Biology (Shanghai, China): Kasumi-1, HL-60, THP-1, U937 and K562. The SKM-1 leukemia cell line was acquired from the Health Science Research Resources Bank (Osaka, Japan), established from a patient with MDS that had progressed to myelomonocytic leukemia.

Bone marrow (BM) samples were obtained from three sAML patients prior to initiation of chemotherapy after obtaining their informed written consent. The samples were enriched for mononuclear cells (MNC) and cultured at 37 °C in RPMI 1640 medium supplemented with 10% heat-inactivated fetal bovine serum (Gibco, MT, USA) in a humidified atmosphere of 5% CO_2_. The collection and analysis of patient samples were approved by the Ethical Committee of the First Affiliated Hospital of Zhejiang University, and written informed consent was obtained from all patients. All methods were carried out in accordance with the approved guidelines and regulations by the First Affiliated Hospital of Zhejiang University. All experimental protocols were approved by the First Affiliated Hospital of Zhejiang University.

### PP2A phosphatase activity assay

1 × 10^6^ SKM-1 cells were seeded into 6-well microtiter plates and treated with different concentrations of LB100 (0, 1.25, 2.5, 5, 10 μM). Following treatment for 6 hours, cells were washed twice with cold water, and lysed in RIPA buffer supplemented with Complete Protease Inhibitor Cocktail (Roche, Mannhein, Germany) for 20 minutes on ice. Cell lysate was sonicated for 10 seconds and then centrifuged at 20,000 g for 15 minutes. Supernatant was then assayed with the PP2A Immunoprecipitation Phosphatase Assay Kit (Millipore, MA, USA).

### MTT assay

Cells were seeded into 96-well microtiter plates (Nunc, Roskilde, Denmark) at densities of either 1 × 10^5^ cells/ml (established cell lines) or 5 × 10^5^ cells/ml (primary AML cells). Cultures were exposed to different drugs for 24 h. After exposure, 20 μl of 3-(4, 5-dimethylthiazol-2-yl)-2, 5-diphenylterazolium bromide solution (MTT, Sigma–Aldrich) was added to each well. The plates were then incubated for 4 h at 37 °C. The MTT-containing solution was then aspirated away, 200 μl DMSO added to each well, and absorbance at 570 nm was measured.

### Assessment of apoptosis

Cells were seeded into 6-well plates, and treated for 24 h at 37 °C with different concentrations of LB100 (0, 1.25, 2.5, 5, 10 μM). After washing with PBS, aliquots of the cells were resuspended in binding buffer, and stained with 5 µl Annexin V and 5 µl propidium iodide (Biouniquer, Nanjing, China) according to the manufacturer’s instructions. Fluorescence-activated cell sorting (FACS) was then performed immediately after staining.

Cells (5 × 10^5^ cells/well) were pre-incubated for 3 h at 37 °C in the presence or absence of 20 μM of the pan-caspase inhibitor z-VAD-fmk (R&D Systems, MN, USA) in DMSO. Cells were then treated with LB100 for 24 h, and processed in the Annexin V-binding assay as described above.

### Hoechst staining

SKM-1 cells were treated with LB100 for 24 h. Cells were then permeabilized with 0.5% Triton X-100 for 30 min, washed with PBS, stained with 10 μg/ml Hoechst for 30 min, and washed with PBS. Nuclear morphology was observed immediately after using a BX51 fluorescence microscope (Olympus, Tokyo, Japan).

### Cell cycle analysis by flow cytometry

Cells were collected after being fixed overnight in 75% ethanol at −20 °C. Fixed cells were washed twice with PBS, then incubated for 30 min with RNase A and propidium iodide (10 μg/ml). Cell cycle analysis was performed using a BDL SRII Flow Cytometer and FACSDiva Software (BD Bioscience, Franklin Lakes, USA). Raw data was analyzed using ModFit LT 3.2 software (Verity Software House, Topsham, USA).

### Leukemia colony-forming assay

LB100-treated SKM-1 cells were seeded in a methylcellulose medium and incubated for 7 d at 37 °C. The number of leukemia colony-forming units (CFU-Ls) containing >50 cells were determined manually under a light microscope (Olympus).

### Western blotting

Cells were washed twice in PBS and lysed in 10 mM Tris, 1 mM ethylenediaminetetra-acetic acid (EDTA), 10 mM KCl, 0.3% Triton, and 0.1 mM phenylmethanesulfonyl fluoride (PMSF). Equal amounts of protein (30–50 μg) were separated on 8–12% SDS-polyacrylamide gels, and transferred to polyvinylidene fluoride (PVDF) membranes. Membranes were blocked with 5% non-fat milk and incubated overnight with the appropriate primary antibody at manufacturer-specified dilutions. Primary monoclonal antibodies were against β-ACTIN (Santa Cruz Biotechnology, CA, USA); CDC25C, p-CDC25C, CDC2, p-CDC2, PP2A-A, PP2A-B, PP2A-C (Epitomics, USA); AKT, p-AKT, PARP, RAS, p-MEK, p-ERK, Raf, p-CamKII, Bcl-2 and p-Bcl-2 (Cell Signaling Technology, MA, USA).

Next, the membranes were washed three times in TBS-T buffer (10 mm Tris–HCl, pH 8, 150 mm NaCl, 0.1% Tween 20), and incubated for 1 h with the corresponding horseradish peroxidase (HRP)-conjugated secondary antibody at 1:5,000 dilution. Bound secondary antibody was detected using an enhanced chemiluminescence (ECL) system (Pierce Biotechnology, IL, USA).

### MicroRNA microarray analysis

Total RNA was extracted from SKM-1 and LB100-treated-SKM-1 cells using the RNeasy mini kit (Qiagen, CA, USA) according to the manufacturer’s instructions. The miRNA microarray analysis was done by KangChen (Shanghai, China).

### RNA extraction and quantitation of miR-181b-1 by real-time quantitative RT-PCR

Total miRNA was extracted from 1 × 10^6^ SKM-1 cells using the RNAiso kit for small RNA (TaKaRa, Japan), and reverse-transcribed using the One Step PrimeScript miRNA cDNA Synthesis Kit (TaKaRa, Japan). The resulting cDNA was quantified using the iCycler Real-time PCR Detection System (BioRad, CA, USA) and SYBR Green (Takara, Japan). The expression of miR-181b-1 was quantified relative to the expression of human U6 small nuclear RNA using the 2^−ΔΔCt^ method. Primers are listed in Table 1.

**Table 1:** The oligonucleotides sequence used in the study

Name	Sequence (5′->3′)
miR-181-b-1 F	AACATTCATTGCTGTCGGTGGGT
U6 F	TGCGGGTGCTCGCTTCGGCAGC
miR-181b-1 precursor F	AATCTCGAGGAACCACAGCTTCCT
miR-181b-1 precursor R	TCCGAATTCACTCCATGTTAGAAC
mutant miR-181b-1 F	GGTCACAATCAGGGAAAGGGAAAGTCGG
mutant miR-181b-1 R	CCGACTTTCCCTTTCCCTGATTGTGACC
Bcl-2 3′UTR F	GGT A ACGCGTCATTATCTTGTCACTG
Bcl-2 3′UTR R	GGGCAAGCTTCTATTTAACTCTGACC
Bcl-2 3′UTR mut1 F	ATTAACTTTGCCCGTGACTCTGTTC
Bcl-2 3′UTR mut1 R	GAACAGAGTCACGGGCAAAGTTAAT
Bcl-2 3′UTR mut2 F	GTTAGACCGTTGCCCATGATATAAAAG
Bcl-2 3′UTR mut2 R	CTTTTATATCATGGGCAACGGTCTAAC

F: forward primer; R: reverse primer.

### *In vivo* tumorigenicity assays

All animal studies were performed according to the guidelines of Animal Care and Use Committee of the First Affiliated Hospital of Zhejiang University and met the NIH guidelines for the care and use of laboratory animals. And all animal studies were approved by IACUC committee of the First Affiliated Hospital of Zhejiang University. Nonobese diabetic/severe combined immunodeficiency (NOD/SCID) mice aged 6 weeks were purchased from the Shanghai Experimental Animal Center of the Chinese Academy of Sciences (Shanghai, China). SKM-1 cells (5 × 10^6^ in 100 μl PBS) were injected subcutaneously into the right flank of each mouse. By 10–14d, when the tumor volumes had reached 90–110 mm^3^, the mice were randomly divided into four groups: DNR + LB100, DNR only, LB100 only, and control. Mice were injected intraperitoneally (i.p.) with 2 mg/kg DNR and/or 2 mg/kg LB100, every other day for a total of 14d. Control mice were injected with an equal volume of PBS. Tumor size was monitored every 3d based on caliper measurements of the two perpendicular diameters; tumor volume was calculated using the formula V = (width^2^ × length × π/6).

### Immunohistochemistry staining

Immunohistochemical staining was performed on the paraffin-embedded sections. Tissue sections were dewaxed and rehydrated before performing antigen retrieval. Anti-BCL-2 (Cell Signaling Technology, MA, USA) were applied at 1:100 dilution in PBS to incubate slides overnight at 4 °C, and incubated with an HRP-conjugated secondary antibody for 1 h at room temperature. DAB was used for color development, and dark brown staining was considered positive. All slides were photographed with optical microscopy Olympus BX51.

### Construction of retroviral vectors and production of ectopic retrovirus

The precursor sequence of miR-181b-1 was PCR amplified from human normal bone marrow mononuclear cells and cloned into MSCVpuro to express miR-181b-1. The mutant miR-181b-1 sequence was created using the primers including the mutated sequences. Primers are listed in Table 1. The MSCVpuro retroviral vector contained a PGK-puromycin-IR ES-GFP (PIG) cassette. The miR-181b-1 precursor sequence or mutant sequence was inserted into the vector between XhoI (CTCGAG) and EcoRI (GAATTC) sites. To produce the ectopic retrovirus, 0.5 × 10^6^ 293T cells were plated in a 60-mm dish the day before transfection. 1.8 μg of retroviral vector DNA and 1.2 μg of PCL-Ampho vector (IMGENEX) were transfected by using the QIAGEN Effectene transfection reagent. Medium was changed with 1 ml of 10% FBS/DMEM after 24 h of transfection. After 48 h of transfection, the virus-containing medium was collected and filtered with a 0.45-μm cellulose acetate (low protein binding) filter.

### Dual luciferase reporter assay

The 3′UTR segment of Bcl-2 containing two predicted target sites of miR-181b-1 was inserted into the downstream of the luciferase reporter in the pMIR-REPORT Dual-Luciferase miRNA Target Expression vector. The mutations were constructed using the primers including the mutated sequences. Primers are listed in Table 1. The pMIR-REPORT vector, pRL-TK vector, and miR-181b-1 retrovirus (or scramble control or mutant miR-181b-1 retrovirus) were co-transfected into 293T cells using the QIAGEN Effectene transfection reagent in 24-well plate. The plasmid pRL-TK containing Renilla luciferase was used as internal control. The 293T cells were harvested after infection for 48 h. The relative luciferase activity was measured by the Dual Luciferase Assay System (Promega, WI, USA).

### Statistical analysis

All data analyses were performed using GraphPad Prism software version 5.0 (GraphPad, CA, USA), and inter-group results were assessed for significance using Student’s t-test. A two-tailed value of P < 0.05 was defined as the threshold of significance.

## Electronic supplementary material

Supplementary information
