# Publisher Correction: PP2A inhibition from LB100 therapy enhances daunorubicin cytotoxicity in secondary acute myeloid leukemia via miR-181b-1 upregulation

**DOI:** 10.1038/s41598-017-14858-z

**Published:** 2017-11-07

**Authors:** Chao Hu, Mengxia Yu, Yanling Ren, Kongfei Li, Dominic M. Maggio, Chen Mei, Li Ye, Juying Wei, Jie Jin, Zhengping Zhuang, Hongyan Tong

**Affiliations:** 1 Department of Hematology, The First Affiliated Hospital, College of Medicine, Zhejiang University, Hangzhou, 310003 People’s Republic of China; 2 Department of Hematology, Hangzhou First People’s Hospital, Hangzhou, 310006 Zhejiang Province People’s Republic of China; 3 Myelodysplastic Syndromes Diagnosis and Therapy Center, The First Affiliated Hospital, College of Medicine, Zhejiang University, Hangzhou, 310003 People’s Republic of China; 4 Department of Hematology, Yin Zhou People’s Hospital, Ningbo, 315040 Zhejiang Province People’s Republic of China; 5 Surgical Neurology Branch, National Institute of Neurological Disorders and Stroke, National Institutes of Health, Bethesda, MD 20892 USA

*Scientific Reports* 7:2894; doi:10.1038/s41598-017-03058-4; Article published online 06 June 2017

The original version of this Article contained an error in the order of corresponding authors. This has now been corrected in the HTML and PDF versions of this Article.

