# Supplementary material for: PP2A inhibition from LB100 therapy enhances daunorubicin cytotoxicity in secondary acute myeloid leukemia via miR-181b-1 upregulation
Source: Sci Rep. 2017 Jun 6;7:2894. doi: 10.1038/s41598-017-03058-4 (PMC5460144; doi:10.1038/s41598-017-03058-4)
Supplement: Supplementary file 1 — Supplementary information [file 41598_2017_3058_MOESM1_ESM.doc]

**Supplementary information**

**PP2A inhibition from ­LB100 therapy enhances daunorubicin cytotoxicity in secondary acute myeloid leukemia via miR-181b-1 upregulation**

Chao Hu^1*^; Mengxia Yu^1, 2*^; Yanling Ren^1, 3^*; Kongfei Li^4^; Dominic M. Maggio^5^; Chen Mei^1, 3^; Li Ye^1, 3^; Juying Wei^1^; Jie Jin^1^; Zhengping Zhuang^5^; Hongyan Tong^1, 3^

^1^Department of Hematology, The First Affiliated Hospital, College of Medicine, Zhejiang University, Hangzhou, 310003, People’s Republic of China

^2^Department of Hematology, Hangzhou First People's Hospital, Hangzhou 310006, Zhejiang, People’s Republic of China

^3^Myelodysplastic Syndromes Diagnosis and Therapy Center, The First Affiliated Hospital, College of Medicine, Zhejiang University, Hangzhou, 310003, People’s Republic of China

^4^Department of Hematology, Yin Zhou People’s Hospital, Ningbo 315040, Zhejiang Province, People’s Republic of China

^5^Surgical Neurology Branch, National Institute of Neurological Disorders and Stroke, National Institutes of Health, Bethesda, MD 20892

*Chao Hu, Mengxia Yu and Yanling Ren contributed equally to this work.

Corresponding author: Prof. Hongyan Tong (zjuhongyantong@163.com) and Prof. Zhengping Zhuang (ZhuangP@ninds.nih.gov)

**Supplementary Figure**


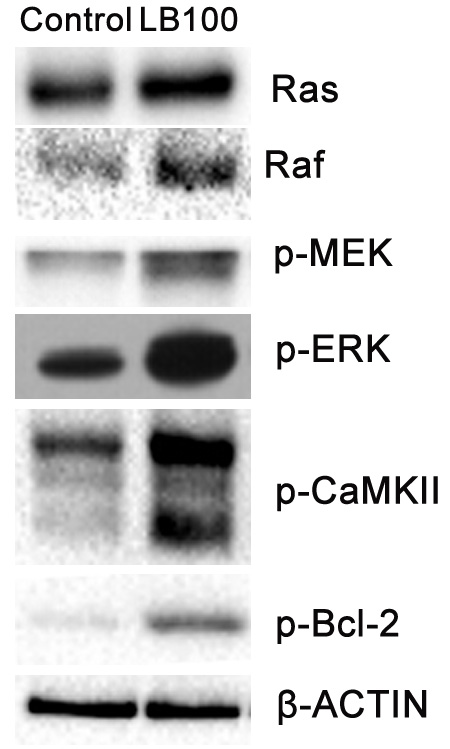


Supplementary Figure S1. Immunoblot demonstrating administration of 5μM LB100 to SKM-1 cells induces increased expression of the RAS/RAF/p-MEK/p-ERK. Also noted is an increased expression of p-CaMKII and p-Bcl-2.
